# The Mast Cell, Contact, and Coagulation System Connection in Anaphylaxis

**DOI:** 10.3389/fimmu.2017.00846

**Published:** 2017-07-26

**Authors:** Mar Guilarte, Anna Sala-Cunill, Olga Luengo, Moisés Labrador-Horrillo, Victoria Cardona

**Affiliations:** ^1^Allergy Section, Internal Medicine Department, Hospital Universitari Vall d’Hebron, Barcelona, Spain; ^2^VHIR Institut de Recerca Vall d’Hebron, Barcelona, Spain

**Keywords:** mast cell, heparin, tryptase, bradykinin, coagulation system, factor XII, fibrinolysis, anaphylaxis

## Abstract

Anaphylaxis is the most severe form of allergic reaction, resulting from the effect of mediators and chemotactic substances released by activated cells. Mast cells and basophils are considered key players in IgE-mediated human anaphylaxis. Beyond IgE-mediated activation of mast cells/basophils, further mechanisms are involved in the occurrence of anaphylaxis. New insights into the potential relevance of pathways other than mast cell and basophil degranulation have been unraveled, such as the activation of the contact and the coagulation systems. Mast cell heparin released upon activation provides negatively charged surfaces for factor XII (FXII) binding and auto-activation. Activated FXII, the initiating serine protease in both the contact and the intrinsic coagulation system, activates factor XI and prekallikrein, respectively. FXII-mediated bradykinin (BK) formation has been proven in the human plasma of anaphylactic patients as well as in experimental models of anaphylaxis. Moreover, the severity of anaphylaxis is correlated with the increase in plasma heparin, BK formation and the intensity of contact system activation. FXII also activates plasminogen in the fibrinolysis system. Mast cell tryptase has been shown to participate in fibrinolysis through plasmin activation and by facilitating the degradation of fibrinogen. Some usual clinical manifestations in anaphylaxis, such as angioedema or hypotension, or other less common, such as metrorrhagia, may be explained by the direct effect of the activation of the coagulation and contact system driven by mast cell mediators.

## Introduction

Anaphylaxis is a severe, life-threatening reaction that results from the systemic effect of mediators and chemotactic substances (1). Pathophysiological mechanisms of human anaphylaxis are not fully understood, but classically mast cells and basophils are considered to play a pivotal role. Allergen crosslinking of specific IgE bound to the high affinity receptor, FcεRI, leads to the activation of mast cells and basophils, inducing cellular degranulation and release of mediators, both preformed or *de novo* synthesized. Mediators of human mast cells comprise pro-inflammatory molecules, such as histamine, leukotriene (LT) B4 and LTC4, or prostaglandin D2 (PGD2), cytokines, vascular endothelial growth factor (VEGF), proteases such as tryptase and chymase, and the highly sulfated polysaccharides, heparin and chondroitin sulfate, being the last two especially abundant in mast cell secretory granules (2). Besides IgE-mediated activation of mast cells and basophils, further mechanisms are involved in the occurrence of anaphylaxis. In mouse models, the role of IgG has been demonstrated. The IgG/antigen complex crosslinking of FcγRIII on macrophages and basophils results in the release of platelet-activating factor (PAF) and the induction of symptoms resembling anaphylaxis (3, 4). Neutrophils have also been suggested as relevant cells in anaphylaxis through IgG1 and 2 in mice (5). In humans, the existence of IgG-mediated anaphylaxis remains unclear. However, studies, showing that PAF— a mediator linked to IgG-mediated anaphylaxis—is essential in human anaphylaxis, reinforce the role of this potential mechanism (6). Recently, the implication of IgG1 and neutrophils in human anaphylaxis has been suggested (7, 8). Other mechanisms linked to severe allergic reactions are the Ig-dependent and independent activation of the complement system, with anaphylatoxin (C3a, C5a) production and binding to their receptors on mast cells, basophils, and other myeloid cells (9, 10) and the direct activation of mast cells by drugs interacting with receptors on these cells.

A myriad of clinical symptoms involving the skin (erythema, itching, urticaria, angioedema), the respiratory tract (bronchospasm, dyspnea, or laryngeal edema), the digestive (diarrhea, vomiting, nausea, pain), or the cardiovascular systems (dizziness and hypotension) can be present during an anaphylactic episode (1). Unusual symptoms include diffuse alveolar hemorrhage, thrombocytopenic purpura, vasculitis, or metrorrhagia, especially in honey bee venom-induced anaphylaxis (11–13). Clinical symptoms of anaphylaxis have classically been attributed to the effects of mast cell/basophil mediators. For instance, histamine binding to its receptors is responsible of pruritus, rhinorrhea, tachycardia, bronchospasm, or hypotension (14) while vasodilation and edema formation, as well as abdominal pain, may be related to tryptase or chymase effects on its target cells (15, 16). On the other hand, mast cell mediators can secondarily promote the activation of different pathways, leading to the release of molecules affecting the clinical expression of anaphylaxis. In this line, it is currently known that the kallikrein–kinin system, the clotting cascade, and the fibrinolytic system may be activated during anaphylaxis (17, 18). The purpose of this review is to give new insights on the implication of the contact and the coagulation systems in anaphylaxis, focusing on the central role of mast cell/basophil mediators on their activation.

## The Contact System in Anaphylaxis

**Figure 1:**
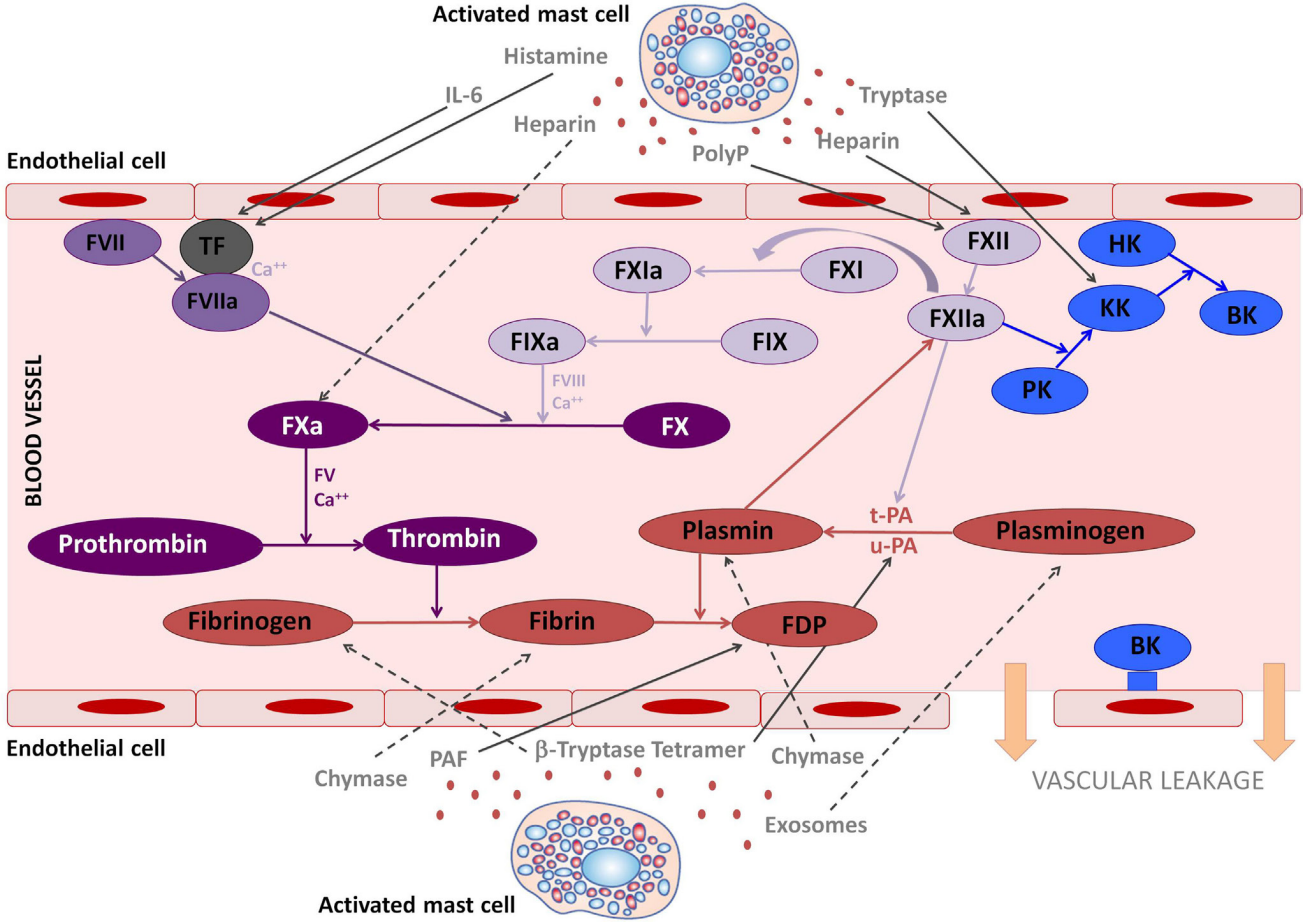
The involvement of mast cell mediators in the coagulation and kallikrein–kinin system. The figure illustrates the effects that mast cells mediators released upon activation during anaphylaxis exert in the kallikrein–kinin, coagulation, and fibrinolytic systems. Solid lines represent activated pathways. Dashed lines are inhibitory pathways. Kinin-forming system factors are represented in blue; the fibrinolytic system is represented in red; the common coagulation pathway in dark purple; the extrinsic coagulation pathway in medium purple; the intrinsic coagulation pathway in light purple. PolyP, polyphosphates; TF, tissue factor; PK, prekallikrein; KK, Kallikrein; BK, bradykinin; HK, high molecular-weight kininogen; tPA, tissue plasminogen activator; uPA, urokinase plasminogen activator; FDP, fibrin degradation products; PAF, platelet-activating factor.

The contact system integrates the plasma bradykinin (BK) formation pathway (the kallikrein–kinin system) and the intrinsic coagulation cascade. In addition, it is involved in thrombus formation, fibrinolysis, and complement activation (19). The contact system is configured by three serine proteases, coagulation factor XII (FXII) or Hageman factor, coagulation factor XI (FXI), and plasma prekallikrein (PK), and by a non-enzymatic cofactor, the high molecular-weight kininogen (HK). FXII, the initiating protein in the kallikrein–kinin system auto-activates to form activated FXII after binding to certain negatively charged surfaces or to macromolecular complexes formed during an inflammatory response or to proteins along cell surfaces (20). There are two plasma substrates for activated FXII, PK, and FXI, and each of these circulates as a complex with HK. Kallikrein is liberated from PK, its plasma precursor, by activated FXII (21). BK, the final product of the system, is released after cleavage of HK by kallikrein (Figure 1). Although the mechanisms of *in vitro* contact system activation have been elucidated, the *in vivo* effects of this complex system in some instances are still unclear.

The effects of BK include peripheral vasodilatation, enhancement of vascular permeability and subsequent vascular leakage, and the contraction of gastrointestinal and uterine smooth muscle, resulting in angioedema, hypotension, and abdominal pain (21), all clinical manifestations of anaphylaxis. The role of kinins in allergy was first established more than 30 years ago from the observation than BK was produced in parallel with histamine release in patients with allergic rhinitis during allergen nasal challenge (22) and in the lungs of asthmatic patients (23), suggesting a link between mast-cell-related conditions and the contact system.

A strong consumption of contact system factors has been observed in patients with anaphylaxis (17, 24, 25) and in IgE-mediated mouse models of anaphylaxis (26). Deficiency or pharmacologic inhibition of FXII, plasma kallikrein, HK, or the bradykinin B2 receptor (BK2R), largely attenuated allergen/IgE-mediated mast cell hyperresponsiveness in mice (17). Interestingly, in this study, both FXII or BK2R-deficient mice, that are resistant to BK signaling, were protected from systemic hypotension during anaphylactic reactions (17), indicating that the contact system is active and contributes to systemic anaphylaxis. In patients with anaphylactic shock to bee venom immunotherapy, intravascular coagulation and diminution of plasma HK has been reported (25) and the sting-challenge test in patients with sting-induced anaphylaxis has been described to promote the generation of FXIIa-C1INH and kallikrein-C1INH complexes, as well as cleavage of HK (24). Recently, activation of FXII and plasma kallikrein has been shown in patients with food or drug-induced anaphylaxis, as well as a 60% cleavage of HK. Furthermore, this activation of the contact system was associated with mast cell degranulation and increased plasma heparin levels (17). Mast cells are an important source of heparin, which contributes to the morphology and storage capacity of their secretory granules (27). *In vivo*, this proteoglycan is exclusively synthesized in mast cells (28). Therefore, heparin derived from human mast cells seems to represent the physiological macromolecule capable of activating the contact system. In experimental models, when heparin either isolated from peritoneal or from human lungs mast cells is added to plasma, contact system activation and BK generation occurs (26, 29). Heparin provides the negatively charged surface for binding and activation of plasma FXII and initiates the kallikrein–kinin cascade. In 2007, several cases of heparin-induced anaphylactic shocks occurred after the use of heparin contaminated with oversulfated condroitin sulfate (30). This contaminated heparin was shown to activate FXII and trigger BK generation (31). Mast cell heparin is a potent FXII activator as it has been observed in mouse models and in humans (22, 32, 33). In acute anaphylaxis, the activation of the contact system correlates with the severity of the episode and the degree of mast cell activation (17).

Tryptase, a trypsin-type serine protease released from mast cells upon activation, may also contribute to kinin production in allergic diseases and in anaphylaxis. Tryptase derived from human lungs was able to release BK mainly through plasma kallikrein activation and to enhance vascular permeability (34). Furthermore, tryptase levels are correlated to plasma HK cleavage during human anaphylaxis (17). Other mast cell mediators potentially capable of activating the contact system are polyphosphates (polyP) and elastase. PolyP are pro-inflammatory agents and potent modulators of the human blood-clotting system, released from activated platelets (35). In mast cells activated through IgE-binding, polyP levels greatly decrease (36). Elastase, a serine protease mainly released by neutrophils but also by basophils and mast cells granules (37), has the capacity to cleave the light chain of HK leaving the kinin sequence untouched and seems to be a positive regulator of the contact system activation (38).

Taken together, these findings indicate that mast cell degranulation during anaphylaxis may trigger FXII activation and the generation of BK through the release of heparin, tryptase and possibly polyP, elastase, or other mediators. However, the role of other mast cell mediators in contact system activation needs to be further explored.

## The Coagulation System in Anaphylaxis

In addition to contact system activation, mast cell mediators may also be involved in the activation of the coagulation system. Hemostasis is maintained by complicated interactions between the coagulation and fibrinolytic systems as well as platelets and vessel walls (39). During anaphylaxis, the release of mast cell mediators may break this hemostasis and the subsequent effects may explain the findings described in these patients.

The finality of the coagulation cascade is to form the clot. It involves the extrinsic, intrinsic, and common pathways. The key initiating factor of the extrinsic pathway is tissue factor (TF), present in the circulation and expressed by cells around the vessels when the endothelial layer is compromised. The intrinsic pathway is activated by FXII, which activates both the contact system *via* PK, and the coagulation system *via* FXI, converting prothrombin to thrombin. Then thrombin further cleaves fibrinogen to insoluble fibrin and activates factor XIII, which will crosslink fibrin polymers (40). Fibrinolysis is activated simultaneously as the coagulation cascade and limits the size of the clot. Plasmin dissolves the fibrin clot into fibrin degradation products. Thus, D-dimers are specific indicators of fibrinolysis. This step is mediated by tissue plasminogen activator (tPA) or urokinase plasminogen activator (uPA) release from vascular endothelium (40) (see Figure 1). The release of tPA is stimulated by tissue occlusion, epinephrine, thrombin, vasopressin, and strenuous exercise (41). Plasminogen is also activated by FXII, although in a weaker manner than tPA and uPA (42). This may suggest a protective role of activated FXII for cardiovascular disease. In addition, plasmin is able to cleave and activate FXII (43), making the interaction between the fibrinolytic and the contact system bidirectional. In fact, angioedema, probably BK-mediated, as a side effect of plasminogen activators administered to patients with thrombotic conditions, has been widely reported (44, 45), indicating contact system activation. Recently the possible role of human tissue mast cells as another important source of tPA has been described (46). Moreover, mast cell tryptase has been shown to activate tPA and pro-urokinase (47, 48).

Few studies have evaluated the interaction of mast cell mediators released during anaphylaxis with the coagulation system. In a case of wasp-sting anaphylaxis, unclottable activated partial thromboplastin time (aPTT) that measures intrinsic coagulation pathway, and a significant anti-Xa activity, with extremely low fibrinogen level with only a slight increase of D-dimer was observed (49). The authors hypothesized that this effects were caused by tryptase tetramers stabilized by heparin released from mast cells during anaphylaxis. On the one hand, heparin acts as an anticoagulant by binding to antithrombin and is responsible of anti-Xa activity and unclottable aPTT. On the other hand, tryptase tetramers act directly on the fibrinolytic pathway by activating pro-urokinase and subsequently degrading fibrinogen. This may explain the low fibrinogen level and the increase in D-dimer reported in the mentioned case (49). In this line, a hyperfibrinolytic state in anaphylactic shock to suxamethonium has been reported (50). Our group also evaluated the coagulation system in anaphylaxis by measuring anti-Xa and aPTT (17). Only 20% of patients had a prolonged aPTT, greater than 100 s, and almost all of them showed a high anti-Xa. These changes occurred concomitantly to HK cleavage. These findings were probably due to mast cell heparin release, which activates FXII leading to the activation of the intrinsic coagulation and the kallikrein–kinin systems, at the same time that it inhibits FXa. Intriguingly, none of these patients developed bleeding (17). In fact, FXII deficiency is an asymptomatic condition and is not associated with bleeding (51). Therefore, it is not surprising that in anaphylaxis, despite activation of FXII usually no hemorrhagic events occur.

The coagulation system has also been assessed in relation to conditions which share some features with anaphylaxis, and in which, mast cells plays a crucial role, such as urticaria. In chronic spontaneous urticaria it was found that coagulation factors (D-dimer, factor VIIa, and fibrinogen) were increased compared to controls and were significantly correlated with disease severity (52).

In a murine model, the tetramer-forming β-tryptases cleaved the α and β-fibrinogen chains and, therefore, the thrombin-initiated clot formation was inhibited (18). This is an additional potential mechanism that could explain why some rare cases of anaphylaxis may develop hemorrhagic disorders. PAF is another mast cell mediator known to interact with the coagulation system. Despite PAF is released by different cell types (neutrophils, endothelial cells, eosinophils, platelets, macrophages, monocytes, and mast cells), in anaphylaxis mast cells are the main source (6). A mice study demonstrated that the release of mast cell PAF could explain disseminated intravascular coagulation symptoms (thrombocytopenia, prolongation of prothrombin time and hypofibrinogenemia, and increase levels of D-dimer), since PAF antagonists could prevent these effects while intravenous PAF was able to reproduce some of the symptoms (53). In addition, another experimental murine study demonstrated that blocking PAF prevents life-threatening peanut-induced anaphylactic reactions (54).

Moreover chymase, a protease exclusively of mast cell origin, has also been shown to affect the coagulation pathway. Mast cell chymase, whose activity depends on heparin (55), is responsible for the degradation and, therefore, inactivation of both thrombin and plasmin, suggesting that mast cell chymase-heparin complexes have a potential function in regulating extravascular coagulation processes, as well as the plasminogen activator/plasmin system (56, 57). Both human chymase and its mouse homolog, mouse mast cell protease-4, had the ability to reduce factor XIIIA, the fibrin-stabilizing factor, levels, and function *via* proteolytic degradation (58). In addition, chymase deficiency led to an increase in the levels and activity of factor XIIIA reducing bleeding times in homeostatic conditions and during sepsis (58).

Histamine has been shown to induce the expression of TF in vascular cells activating the H1, but not the H2, receptor (59, 60), revealing the potential effect of histamine on the extrinsic coagulation pathway. Some newly generated cytokines from mast cells, such as IL-6, also affect the coagulation system. It has been described that IL-6 may amplify activation of coagulation through upregulation of TF (61). Finally, it has also been shown that mast cell-derived exosomes activate endothelial cells to secrete plasminogen activator inhibitor type 1 and induce procoagulant states (62).

Thus, as it occurs with the contact system, several mast cell mediators have a potential role in the regulation of coagulation and fibrinolysis. When a massive release of mast cells mediators occurs, as in anaphylaxis, the activation of these two systems may contribute to the pathophysiology of the multisystemic reaction. In addition, depending on the relative release of each mediator, the clinical effects may differ.

## Biomarkers to Assess the Contact and Coagulation System Involvement in Anaphylaxis

Currently, the only biomarkers that can be measured routinely in anaphylaxis are histamine and tryptase. Reliable biomarkers to assess contact system activation and the generation of BK have not been established.

Measurement of BK *in vivo* is practically impossible due to its rapid degradation by kininases once released. As BK is present in the circulation for only few seconds, the measurement of activation products of the contact system can be valuable biomarkers as they may reflect recent BK production. Thus, we speculate that the most suitable biomarkers to assess contact activation are circulating cleaved HK and kallikrein activity but these measurements are very labor intensive since standardized assays are lacking. To assess the coagulation system, the observation of prolonged aPTT reveals activation of the intrinsic coagulation pathway. Increased levels of anti-Xa reflect the possible effect of heparin and the inhibition of the common coagulation pathway. Regarding the fibrinolytic system, increased levels of plasmin complexes and also increased levels of D-dimer, in patients without a thromboembolic event, should raise the suspicion that the contact and fibrinolytic systems are activated.

## Conclusion

Anaphylaxis is a complex allergic reaction where multiple biological systems are involved. Further mechanistic studies to discern the involvement of molecules from the contact and coagulation systems are warranted to completely understand the pathophysiology and subsequent clinical effects during anaphylaxis. In order to assess all these complex connections between mast cell mediators and the activation of the contact and coagulation systems, a wide array of potential biomarkers are needed and should be monitored at multiple time-points together with their functional effects. Only then, will we be able to have a complete overview of the interactions and subsequent effects of each mediator and pathway, and maybe also offer a closer insight to new potential diagnostic markers or therapeutic targets for anaphylaxis.

## Author Contributions

All authors made substantial contributions to conception and design of the review. MG and AS-C drafted the manuscript. ML-H, OL, and VC participated in revising it critically for important intellectual content. All authors approved the final version of the manuscript.

## Conflict of Interest Statement

The authors declare that the research was conducted in the absence of any commercial or financial relationships that could be construed as a potential conflict of interest.
